# Study protocol for a randomised trial of nicotine-free cigarettes as an adjunct to usual NRT-based cessation practice, in people who wish to stop smoking

**DOI:** 10.1186/1471-2458-11-37

**Published:** 2011-01-14

**Authors:** Natalie K Walker, Colin Howe, Chris Bullen, Michele Grigg, Marewa Glover, Hayden McRobbie, Murray Laugesen, Stephen Vander Hoorn, Robyn Whittaker

**Affiliations:** 1 Clinical Trials Research Unit, School of Population Health, The University of Auckland, Private Bag 92019, Auckland 1142, New Zealand; 2 Previous Address: The Quit Group, PO Box 12605, Wellington, New Zealand; 3 Current Address: Litmus, Wellington, New Zealand; 4 Centre for Tobacco Control Research, Social & Community Health, School of Population Health, The University of Auckland, Private Bag 92019, Auckland 1142, New Zealand; 5 Previous Address: Clinical Trials Research Unit, School of Population Health, The University of Auckland, Private Bag 92019, Auckland, New Zealand; 6 Current Address: Queen Mary University of London, Wolfson Institute of Preventive Medicine, Barts and The London School of Medicine and Dentistry, Charterhouse Square, London, EC1M 6BQ, UK; 7 Health NZ Ltd., 36 Winchester St, Lyttelton, Christchurch 8082, New Zealand; 8 Statistics Department, The University of Auckland, Private Bag 92019, Auckland 1142, New Zealand

## Abstract

**Background:**

Current smoking cessation treatments focus on addressing the pharmacological dependence of smokers on nicotine. However, new strategies are needed that address both nicotine dependence and the psychological dependence on cigarettes as the source of nicotine. Evidence from a number of small smoking cessation studies suggests that the use of cigarettes with reduced nicotine content, in combination with nicotine replacement therapy (NRT), may help reduce withdrawal symptoms and increase quit rates. This paper describes the protocol for a large randomised-controlled trial to test the effect of using nicotine-free cigarettes together with NRT on long-term quit rates.

**Methods/design:**

This single-blind, randomised trial aims to recruit 1,410 participants through the national telephone-based Quitline service in New Zealand. Participants in the treatment arm will be asked to stop smoking nicotine-containing cigarettes on their chosen Quit day and smoke *ad libitum *nicotine-free (Quest 3) cigarettes for six weeks. At the same time people in this group will be asked to start using NRT patches, gum and/or lozenges (as recommended by Quitline) for eight weeks. Participants in the control arm will be asked to stop smoking completely on their chosen Quit day and start using NRT patches, gum and/or lozenges (as recommended by Quitline) for eight weeks. Data collection will occur at baseline, three and six weeks, and three and six months after Quit day. The primary outcome is the proportion of participants who self-report seven-day point prevalence abstinence at six months since Quit date.

**Discussion:**

Smoking prevalence in New Zealand has changed little in recent years (particularly in Māori, the indigenous people of New Zealand) and additional options for smokers who want to quit are needed. Although a variety of methods are available to help, many are expensive, have side effects, and despite their use most quit attempts still fail. This trial will test the balance of benefits and risks of a new strategy for people to overcome nicotine dependence. Since smoking is the leading cause of lost healthy life years in New Zealand, if proven effective this strategy is likely to have substantial public health benefits.

**Trial registration:**

Australia and New Zealand Clinical Trials Register (ANZCTR): ACTRN12608000410358

## Background

Smoking is the leading cause of lost healthy life years in New Zealand. In 1990, 28% of New Zealand adults smoked, with this figure dropping to 24.9% in 1996/7, 24.5% in 2002/3 [1] and 19.7% in 2006/7 [2]. In Māori (indigenous New Zealanders) there has been little downward trend in smoking prevalence since the early 1990's, with 45.5% of Māori smoking in 1996/7, and 47.2% smoking in 2002/3 [2]. However, in 2006/7 there was a sharp fall in smoking prevalence in Māori, down to 38% [2]. Despite this recent evidence of change, based on the current rate of progress it is estimated that it will take 100 years before the adult smoking rates in New Zealand reach 3.6%, the level of all New Zealand doctors now smoking [3]. New approaches to smoking cessation are urgently needed, ideally ones that give additional benefits to existing strategies. One approach that warrants further investigation is to look at ways of reducing the association between smoking and nicotine delivery.

Nicotine is the constituent of tobacco principally responsible for the addictive nature of cigarette smoking. Reward pathways in the brain are stimulated within two to three minutes of inhalation. The smoker is compelled to continue dosing throughout the day to maintain the desirable effects of nicotine and mitigate feelings of withdrawal [4]. Other factors, besides nicotine, may also play a part in the dependency-forming aspects of smoking, including the effects of other chemicals in tobacco smoke, the strong behavioural/tactile association of smoking and the social pleasure of smoking.

There is some evidence that progressive reduction in the level of nicotine in cigarette tobacco can reduce the level of nicotine dependence in smokers, with minimal compensatory smoking and without any negative health effects [5]. Reduced nicotine cigarettes have been produced in the past (as the Quest brand marketed by Vector Tobacco Inc, USA), with the tobacco altered through genetic modification. Quest 1, 2 and 3 cigarettes have a nicotine yield of 0.6 mg, 0.3 mg and ≤0.05 mg per cigarette respectively, an average nicotine content of 8.9 mg, 5.1 mg and 1.0 mg per cigarette respectively, and a tar content of 10 mg per cigarette (similar to the level in regular cigarettes). These cigarettes are no longer commercially available, but are available for research purposes. The use of Quest 3 cigarettes, so called nicotine-free cigarettes, warrant further scientific investigation, as current evidence suggests that the use of nicotine replacement therapy (NRT) in combination with such cigarettes may have a greater impact on the signs and symptoms of withdrawal and cessation rates, compared to the use of nicotine-free cigarettes alone [5]. However, there is a need to undertake adequately powered clinical trials to clarify the true strength and direction of the above association, with at least six months follow-up data collected.

## Methods/design

### Objectives

The primary objective of this trial is to determine the combined effect of nicotine-free cigarettes and NRT (used immediately after quitting), on long-term quit rates. Secondary objectives include whether such an intervention is cost effective and acceptable.

### Study design

This study is a parallel, randomised controlled clinical trial, with allocation concealed from the study researchers.

### Study population

The study population will be registered and eligible callers to the national toll-free Quitline cessation service.

### Inclusion criteria

Participants will be smokers from throughout New Zealand who want to stop smoking. Participants will be eligible provided they: are at least 18 years of age, have their first cigarette within 30 minutes of waking, are able to provide verbal consent, and have a telephone.

### Exclusion criteria

Pregnant women and women who are breastfeeding will be excluded from the trial. People will also be excluded from the trial if they meet any of the following criteria: current users of NRT products; current clients of Txt2Quit and NRT Online (current Quitline services); current users of non-nicotine based cessation therapies (e.g. buproprion, clonidine, nortriptyline or varenicline); they only use non-cigarette tobacco products (e.g. pipes, cigars); they have had a myocardial infarction within the last two weeks; and have had angina, severe cardiac arrhythmia or a stroke in acute phase within the last two weeks. The medical conditions above are listed contraindications for the use of NRT patch, gum, and lozenge. A number of disease conditions are known where precautions for the use of NRT products are indicated, such as severe cardiovascular disease (occlusive peripheral arterial disease, cerebrovascular disease, stable angina pectoris, and uncompensated heart failure) and vasospasm, uncontrolled hypertension, renal or hepatic impairment, active duodenal ulcers and gastric ulcers. Quitline staff will ask potential participants about these conditions, prior to participant randomisation.

### Randomisation: allocation concealment and sequence generation

All potential participants will be assigned a unique registration number allocated by a central computer following details submitted on a web-based form. This number will be used to identify each randomised participant who has consented to take part. Participants will be randomised by computer, with stratified minimisation by sex, ethnicity (Māori, non-Māori), and level of nicotine dependence (> 5 points, ≤ 5 points on the Fagerström Test of Nicotine Dependence (FTND) Questionnaire [6]), to ensure a balance in these key prognostic indicators between the intervention and control group.

### Blinding

Due to the nature of the intervention only single blinding (of researchers, not of participants) is possible in this trial. Members of the trial steering committee, management committee, and other team members from the Clinical Trials Research Unit (with the exception of the project co-ordinator and the Quitline research manager) will remain blinded to treatment allocation until the code is broken (after the last follow-up call is completed and the data recorded). The project co-ordinator will not be blinded as they will be responsible for distributing the nicotine-free cigarettes to participants. The research manager and research assistants based at Quitline will not be blinded to treatment allocation, as they are required to know which group each participant has been allocated to in order to conduct the scheduled follow-up calls.

### Study intervention

Participants will be randomised to a six week intervention of nicotine-free cigarettes (Quest 3) plus eight weeks of usual care (NRT plus behavioural support) *versus *eight weeks of usual care (NRT plus behavioural support).

**Figure 1:**
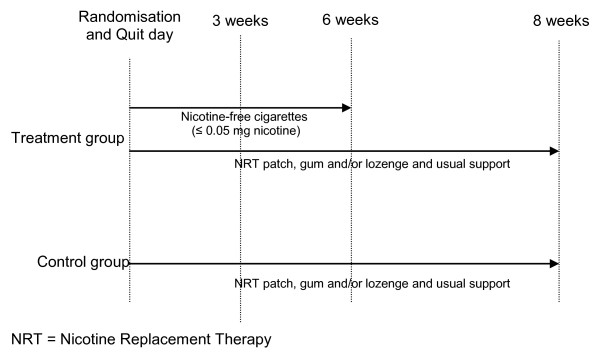
**Schematic of treatment allocation during intervention period**.

• Treatment group: Participants allocated to the treatment arm will be asked to stop smoking their normal cigarettes on their chosen Quit day and switch to the nicotine-free cigarettes (Quest 3) couriered to them, then at six weeks to stop smoking the nicotine-free cigarettes (Figure 1). Participants will be told that they should try all the techniques suggested by the Quitline advisors to help them get through the withdrawal period (i.e. drink water, deep breathe, do something else, delay), but if they still feel they must have a cigarette, they should only smoke the nicotine-free cigarettes provided. Participants will also be supported by Quitline in the usual way for eight weeks, with counselling/advice and NRT in the form of a patch, gum and/or lozenge (Figure 1). The Quest 3 cigarettes used in this trial have recently been reformulated by Vector Tobacco Inc and no longer contain genetically modified tobacco. The "new" cigarettes still have a similar nicotine content (1.5 mg per cigarette) and yield (≤ 0.05 mg per cigarette) as the original Quest 3 cigarettes, but the tar content has been reduced from 10mg to 4mg per cigarette. In order to verify the nicotine and tar content of the trial cigarettes, independent verification will be undertaken by Labstat Canada. Specifically, the nicotine content of the unburnt nicotine-free cigarettes will be assessed (including analysis of nicotine alkaloids by gas chromatography and tobacco moisture), as will total particulate matter, water, nicotine, particulate matter (water and nicotine free), carbon monoxide and puff number.

• Control group: Participants allocated to the control arm of the trial will be asked to stop smoking completely on their chosen Quit day and will be supported by Quitline in the usual way for eight weeks, with counselling/advice and NRT in the form of a patch, gum and/or lozenge (Figure 1).

Current standard cessation practice at the New Zealand Quitline (as at December 2010) is as follows (other cessation providers in New Zealand do not follow the process outlined here): Quitline issues by post, a Quit Card (with a 90 day expiry) for eight weeks supply of up to three NRT products (patch, gum and/or lozenge) and makes routine support calls by telephone over eight weeks. These support calls are administered by the Quitline advisors as required. Quitting support generally involves an average of three follow-up telephone calls, each lasting about 10-15 minutes. However, if people do not wish to receive support calls, they are not scheduled but callers are advised that they can phone Quitline at any time for support. Participants are advised to take the Quit Card to a pharmacist in exchange for subsidised patches, gum and/or lozenges (NZ$3 per item per four-week course of NRT). The strength of NRT patch, gum, and/or lozenges to be used by each participant is determined by the Quitline advisor (as per their dosage guidelines) as to the degree of each person's nicotine dependency. "Quit day" in this study will refer to the day each participant stops smoking cigarettes containing nicotine.

### Baseline assessments

Participants from throughout New Zealand will call Quitline seeking advice and wishing to enrol in the Quitline smoking cessation programme. A Quit advisor from Quitline will undertake the standard Quitline assessment. A list of those individuals who indicate that they wish to take part in research will be transferred from the Quitline database to the trial research assistants based at Quitline. The research assistants will attempt to make contact with people on the list. Those contacted will be told about the trial and asked if they would like to participate. If the potential participant indicates that they are interested, they are consented and asked for demographic data (age and sex) and checked to see if they meet the inclusion criteria for the trial. For those that do meet the inclusion criteria, further details will be collected about the participant's ethnicity and level of nicotine dependence (as determined by the FTND Questionnaire [6]) and they will be randomised Data from this initial assessment will then be electronically transferred (securely) to the Clinical Trials Research Unit's Oracle database. The research assistant will then collect the following baseline data from each participant over the telephone.

• Demographic information: Socio-economic position, based on income, education and occupation.

• Smoking history: Age when started, number of cigarettes smoked per day, number of years as regular smoker, number of previous unsuccessful attempts to give up in past 12 months and the method used, other types of smoking (pipe, cigar), usual brand/pack size (and how long pack lasts), and type of cigarettes smoked per day (e.g. roll-your-own or factory-made).

• Other smoking related information: Self-rated chances of quitting (measured on a scale of one to five, where one is very low and five is very high), household smoking, smoking in cars.

• Concomitant medication: Information about types of medication currently used will be collected.

• The physical signs and symptoms associated with withdrawal: Measured using the Mood and Physical Symptoms Scale (MPSS) [7]. Additional withdrawal questions will also be asked related to the frequency of disturbed sleep, anxiety, mouth ulcers, cough, impatience, dizziness and increased dreaming [8].

• Alcohol use and abuse: Measured using the Alcohol Use Disorders Identification Test (AUDIT-C), a screening tool that helps to identify people who are hazardous drinkers or have active alcohol use disorders (including alcohol abuse or dependence) [9].

### Outcome measures

#### Primary outcome

The proportion of participants who self-report having stopped smoking at six months after their Quit date. 'Stopped smoking' will be defined as having consumed no nicotine cigarettes (not a single puff) in the previous seven days (seven-day point prevalence).

#### Secondary outcomes

The following secondary outcome measures will be assessed at three and six weeks after the Quit date in the treatment group only:

• Information related to the use of the nicotine-free cigarettes: Participants will be asked for their views on the use of the nicotine-free cigarettes (including concerns, whether they liked the taste of them, etc), and whether the six weeks use of the cigarettes was adequate. The number of nicotine-free cigarettes and normal cigarettes smoked each week during the intervention period will also be recorded.

• Information related to the use of the NRT: Participants will be asked about their use of NRT during the treatment period.

The following secondary outcome measures will be assessed in all participants at three weeks, six weeks, three months and/or six months after the Quit date:

• The physical signs and symptoms associated with withdrawal at three and six weeks: Measured using the Mood and Physical Symptoms Scale (MPSS) [7]. Additional withdrawal questions will also be asked related to the frequency of disturbed sleep, anxiety, mouth ulcers, cough, impatience, dizziness and increased dreaming [8].

• Self-rated chances of quitting at three and six weeks: Measured on a scale of one to five, where one is very low and five is very high.

• Proportion of participants who have stopped smoking at three weeks, six weeks and three months: Defined as no nicotine cigarettes (not even a puff) in the past seven days.

• Continuous abstinence: Defined as self-report of smoking no more than five nicotine cigarettes from the Quit date, collected at all time points.

• Proportion of participants who have significantly reduced daily smoking level at six months: Defined as reducing consumption by at least 25% (in terms of numbers of cigarettes per day or weight of loose tobacco per day).

• Number of cigarettes currently smoked per day: If the participant is still smoking (all times points). Pack size (and how long pack lasts) if smoking roll-your-owns.

• Cost information: Cost outcomes will include cost per quitter, cost per person reducing their daily cigarette consumption, and cost per Quality Adjusted Life Year of life saved among quitters. The tobacco expenditure savings to individual smokers will also be calculated using data on the daily amount smoked prior to quitting and the price of the particular products smoked.

• Use of NRT: Participants will be asked about continued use of any NRT (including type, dosage, flavour, and frequency of use) at all time points.

• Use of non-NRT methods of cessation at six months: Participants will be asked about their use of non-NRT methods of cessation such as buproprion, clonidine, nortriptyline, varenicline, acupuncture etc.

• Alcohol use and abuse at six months: Measured using the AUDIT-C [9].

• Serious adverse events: Information regarding any serious adverse events and whether they are related to treatment will be collected at all time points.

• Concomitant medication: collected at all time points

### Sample size

A sample size of 1,410 people (705 in each group, with a loss to follow-up rate of 20% and at least 25% Māori in the sample) is required for this trial. This number will provide 90% power at p = 0.05 to detect a difference in point prevalence abstinence of 7.5% at six months. In addition, this sample size will allow the consistency of effects for pre-specified subgroups to be assessed. It is expected to take 18 months to recruit the required sample size, based on a previous cessation trial run through the New Zealand Quitline [10].

### Withdrawal criteria

If, at any time after randomisation, significant intolerance to the study treatment is suspected, the treatment (or control) can be withdrawn. Should participants require discontinuation of study treatment for any reason (see below), or if they elect to cease taking treatment, follow-up calls and data collection will continue as scheduled as if they were continuing with the randomised treatment. Participants may have the study treatment withdrawn if one or more of the following occurs:

• The participant makes a voluntary decision to withdraw from follow-up, or from the treatment.

• The participant has any serious clinical adverse event, intercurrent illness, or other medical condition that indicates to the principal investigator that continued treatment with the study treatment is not in the best interest of the participant. The study treatment will be withdrawn if the participant develops any life threatening or seriously disabling illness or is admitted to hospital.

• The participant becomes pregnant during the course of the trial. Such participants will be asked to discuss on-going NRT use with their general practitioner or lead maternity caregiver.

• The investigator feels that it is in the best interests of the participant.

• The study is terminated.

If the participant discontinues treatment due to a serious adverse event, the participant will be followed until the event resolves or there is a return to a clinically acceptable medical status. Participant deaths or serious adverse events, which occur within 30 days of discontinuation, will be reported to the project co-ordinator.

### Data management

The design and management of all databases associated with this trial will be undertaken by the data management and information technology groups at the Clinical Trials Research Unit. Data will be stored in the Unit's Oracle database and linked to the electronic Case Record Form (CRF) data where applicable. Validation rules for each CRF will be specified by the project co-ordinator, in association with the senior data manager. These rules will include range checks so that inaccuracies in data collection can be identified early. A query will be raised as soon as any values are entered that are outside the allowed range or if data are missing. The research assistants at Quitline will amend the form as soon as a query is raised.

### Data monitoring

An independent person will monitor the trial conduct. This monitor will audit Quitline and the Clinical Trials Research Unit during the trial to ensure that the study protocol is being adhered to. At Quitline the monitor will audit every randomised participant's records to ensure their existence, that they meet the inclusion criteria and have provided informed consent, and that the treatment is being distributed within the limits of the protocol. The monitor will review the study documentation and records held by Quitline to ensure that documentation is up-to-date and record keeping meets the requirements specified in the protocol and complies with regulatory requirements. The monitor will visit Quitline early on during the study (after ten participants have been randomised), at study close-out and twice during the course of the trial. The monitoring task for the trial is unlikely to be onerous as the majority of data for this trial will be collected electronically, with range and logic checks built into the programme used. In the rare event that the computer programme is not accessible (e.g. power cut, system down), hard copies of the CRF's will be completed by the research assistants at Quitline, with the data entered onto the Unit's computer database at a later stage. Hard copies of the CRF's will be checked for consistency by the monitor and checked against the electronic copy. The monitor will audit the Unit every three months to see that the product supply records are in order and that there are sufficient supplies remaining, that the nicotine-free cigarettes are being stored appropriately and that the handling of unused nicotine-free cigarettes complies with study procedures. A Data Safety and Monitoring Committee has not been established for this trial as the trial does not meet two or more of the criteria stipulated by Ellenburg el al (2002), for setting up such a committee [11].

### Data analyses

This trial has been designed with the assistance of a senior statistician at the Clinical Trials Research Unit, who will continue to advise and assist with analyses of the trial data.

#### Treatment effects

Analyses will be undertaken on an intention-to-treat basis, according to a pre-specified statistical analysis plan using SAS version 9.1.3. A per-protocol analysis will also be performed in order to check the robustness of the results. No interim analysis will be undertaken. Chi-squared analyses will be used to compare the proportion quit by treatment group, and incidence rates, relative risks, risk differences, 95% confidence intervals and two-sided p-values will be calculated. Logistic regression analyses will be utilized to adjust for important prognostic factors. Continuous outcome data will be analysed using multiple linear regression modelling. Both the number of cigarettes per day and withdrawal symptoms over time will be compared using analysis of covariance. Pre-specified subgroup analyses using tests for heterogeneity will be undertaken for ethnicity (Māori, non-Māori), age (< 40 years, ≥ 40 years), sex and socioeconomic status (dichotomised as those who left school below Year 12 or with no school qualification, and those who completed Year 12 and above). Analyses will also be undertaken to examine time-to-first-lapse using Kaplan-Meier analysis and self-reported seven-day point prevalence of smoking abstinence at six months in those with complete smoking status data.

#### Cost analyses

Costs analyses will be undertaken if a statistical difference between the groups is observed for the primary outcome of interest. Cost outcomes will include cost per quitter, cost per person reducing their daily cigarette consumption, and cost per quality adjusted life year of life saved among quitters (using discount rates of 0%, 5% and 10%). These data will then be compared with New Zealand data from Quitline and other NRT service providers, in addition to information from various international studies. This modelling will take a health sector perspective. However, the tobacco expenditure savings to individual smokers will also be calculated (for those who quit and cut down) to give a more societal perspective on the benefits (especially to low-income smokers). This calculation will use data on the daily amount smoked prior to quitting and the price of the particular products smoked. For those who cut down their consumption by a significant margin (i.e. 25% or more), the cost per person reducing their daily cigarette consumption will be calculated. Different approaches to assessing the benefit of years per life saved - including the use of New Zealand specific data on the relative risk of death as per Hunt et al 2005 [12], will also be explored.

#### Tolerability

All randomised participants who receive at least one dose of study treatment or control will be included in the planned analyses. Comparison of the frequency of treatment withdrawal between the treatment and the control group will be tested using Chi-square statistics. The numbers of participants discontinuing treatment prematurely for any reason will be summarised by treatment group and by reasons for discontinuation. Serious adverse events will be coded using ICD-10 AM codes. The incidence of all suspected serious adverse treatment reactions will be summarised by treatment group.

#### Procedures to account for missing data

For treatment effects, sensitivity analysis will be carried out to determine the effect of missing data (such as participants lost to follow-up). All participants lost to follow-up will be presumed to be smoking [13].

### Ethics

Ethics approval has been granted from the National Multi-Regional Ethics Committee (Ethics number MEC/08/10/117). Maintenance of confidentiality and compliance with New Zealand's Privacy Leglisation will be emphasised to all study participants. Participation in the study is entirely voluntary. Verbal consent will be obtained at the time of contact with Quitline, however a written consent form and patient information sheet will be posted out to participants for their information. Data will be entered, stored and backed-up in a secure manner via the Unit's internet data management system. Participants will be acknowledged in all publications and presentation of the results.

## Discussion

Increasing quit rates would have a significant positive impact upon the health and well-being of smokers and their households [14]. Unfortunately many smoking cessation aids on the market are relatively expensive, have side effects and are only moderately efficacious. There would be great interest in a novel smoking cessation aid that may be an addition to current therapies or indeed could be used by those who do not want to or can't use NRT or other cessation pharmacotherapies. Initial studies have shown that reduction in exposure to nicotine in cigarettes can reduce the level of nicotine dependence in smokers, at least in the short term, and that combined use of reduced nicotine cigarettes and NRT appears more effective at increasing quit rates than using reduced nicotine cigarettes alone or NRT alone [5]. However, larger and more long-term studies are still needed to clarify the strength of this relationship.

If the combined use of nicotine-free cigarettes and NRT is shown to have a positive impact on smoking cessation and is cost effective, further research would focus on the implications of the research findings on policy. Smoking prevalence has changed little in recent years in New Zealand. Clearly the current range of smoking cessation aids are not having a strong enough impact upon smoking cessation rates and consequently additional options for smokers who want to quit are needed. This trial will test the balance of benefits and risks of a new strategy for people to overcome nicotine dependence. Since smoking is the leading cause of lost healthy life years in New Zealand [1] if proven effective this strategy has the potential to have substantial public health benefits.

## Competing interests

The nicotine-free cigarettes (Quest 3) used in this trial have been purchased from Vector Group Ltd, USA. NRT patches, gum and/or lozenges used by all study participants after Quit day are purchased by participants (using the subsidized Quit Card they receive from Quitline) from community pharmacies. No authors have received support from any companies for the submitted work. No authors have any relationship with Vector, a company that might have an interest in the submitted work. HM has received honoraria for speaking at research symposia and received benefits in kind and travel support from, and has provided consultancy to the manufacturers of smoking cessation medications. CB and HM have previously undertaken research on behalf of NicoNovum (a manufacturer of smoking cessation medications), but prior to the purchase of the company by RJ Reynolds. NW has provided consultancy to the manufacturers of smoking cessation medications, received honoraria for speaking at a research meeting and received benefits in kind and travel support from a manufacturer of smoking cessation medications. MG has provided consultancy to the manufacturers of smoking cessation medications. The author's spouses, partners, or children have no financial relationships that may be relevant to the submitted work. All authors have no non-financial interests that may be relevant to the submitted work.

## Authors' contributions

NW, CB, MGr, MGl, HM, ML, SVH, and RW conceived the original idea for the trial, sought funding and wrote the protocol. CH and MGr manage the day to day running of the trial, including participant follow-up. This protocol paper was written by NW, with input from all co-authors. All authors read and approved the final manuscript. NW will act as guarantor for this paper.

## Pre-publication history

The pre-publication history for this paper can be accessed here:

http://www.biomedcentral.com/1471-2458/11/37/prepub
